# The impact of *ABCB1* gene polymorphism and its expression on non-small-cell lung cancer development, progression and therapy – preliminary report

**DOI:** 10.1038/s41598-020-63265-4

**Published:** 2020-04-10

**Authors:** Izabela Zawadzka, Agnieszka Jeleń, Jacek Pietrzak, Marta Żebrowska-Nawrocka, Katarzyna Michalska, Dagmara Szmajda-Krygier, Marek Mirowski, Mariusz Łochowski, Józef Kozak, Ewa Balcerczak

**Affiliations:** 1 Laboratory of Molecular Diagnostics and Pharmacogenomics, Department of Pharmaceutical Biochemistry and Molecular Diagnostics, Medical University of Lodz, ul. Muszynskiego 1, 90-151 Lodz, Poland; 2 Department of Thoracic Surgery, Memorial Copernicus Hospital, Medical University of Lodz, Lodz, Poland

**Keywords:** Cancer, Genetics, Molecular biology, Biomarkers, Medical research, Molecular medicine

## Abstract

The *ABCB1* gene belongs to ATP binding cassette (ABC) transporter genes that has been previously implicated in cancer progression and drug response. This study aimed to evaluate the association between the SNP 3435 and the expression of the *ABCB1* gene in lung cancer patients in the Polish population in comparison to clinicopathological parameters and treatment. 150 RNA and 47 DNA samples were isolated from 49 lung cancer cases including both tissue samples and blood taken from the same patients at three time points: diagnosis, 100 days and one year after the surgical intervention. Qualitative and real-time PCR analysis of expression were done, also genotyping by PCR-RFLP. Mutant homozygous TT and allele T are present statistically significantly more frequently in the group of patients with lung cancer. There is no difference with expression level in lung cancer tissue and blood sample taken from the same patients before surgical treatment. On the basis of blood samples analysis it was observed that the expression level of *ABCB1* mRNA was growing in time. Higher levels were marked after 100 days and one year after the surgical intervention. The complementary pharmacological treatment induced higher expression levels of *ABCB1*. The presented data suggest an important role of *ABCB1* in lung cancer, the increasing level of *ABCB1* mRNA which can be connected with induction of multidrug resistance mechanism is also significant, that observation must be confirmed in further analysis.

## Introduction

Malignant tumors after a cardiovascular disease are the leading cause of death in Poland. Lung cancer is the second most common cancer in men and women, representing approximately 13% of all new cancers. Among all cancers, lung cancer is responsible for the largest number of deaths in oncology patients in highly developed countries. Recent studies suggest that it can overtake breast cancer as the leading cause of cancer deaths among women in Europe by the middle of this decade. According to the estimates of the Polish Oncology Society report “Current state of cancer control in Poland” from 2014, in 10 years’ time the number of deaths from lung cancer may amount to over 30.000 cases annually^1^.

Despite the alarming statistics of the incidence and mortality due to lung cancer, the development of effective therapy remains unattainable. Most patients diagnosed with lung cancer already have an advanced disease − 40% is stage IV and 30% is stage III^2^. The five-year survival rate of NSCLC varies from 73% in early detection to 3.7% in advanced metastatic disease^3^. Understanding the basic biological and molecular mechanisms of developing lung cancer contributed to the development of personalized medicine. A simple binary division of lung cancer into non-small cell lung cancer (NSCLC) and small-cell lung cancer (SCLC) is no longer relevant as knowledge about the human genome is constantly increasing. The ability to genotype creates the possibility of an individual analysis. Lung cancer is currently divided into molecular subtypes and targeted therapies may affect the effectiveness of chemotherapy, and extend the time free from progression and overall survival^4^.

The ABC transporters are a superfamily of transmembrane proteins that transport many different substrates across lipid extracellular and intracellular membranes metabolites, carcinogens and cytotoxic drugs including anticancer drugs^5^. In the human genome, 48 different ABC transporters were identified and divided into seven subfamilies A-G based on sequence similarities^6^.

The *ABCB1* gene, encoding the P-glycoprotein, is located on chromosome 7q21.1, consists of 28 introns and 28 exons. *ABCB1* mRNA is 4.7 kb and is contained in the coding region of 120 kb. The *ABCB1* gene has been extensively studied for characteristic polymorphisms and about 50 SNPs for *ABCB1* have been identified^7^. The most frequently studied polymorphisms in the *ABCB1* gene are C1236T (rs1128503), G2677T/A (rs2032582) and C3435T (rs1045642). Genetic variants associated with a change in the amount or activity of transport proteins lead to the loss of the physiological role of these proteins and altered various drugs transporter’s functions^8,9^. These three most common *ABCB1* SNPs in the Caucasian population have been found to be in linkage disequilibrium. The allelic frequencies of these three SNPs are highly variable between ethnic groups^10^.

The most widely studied variant of *ABCB1* is a commonly synonymous C to T transition at nucleotide position 3435 in exon 26 (3435 C > T). Although this transition does not change its encoded amino acid with Ile at position 114522, TT variant has been significantly associated with the decreased mRNA expression and protein stability and may have reduced the drug transport capacity^10^. So far the effect of synonymous polymorphisms on the protein has not been fully understood. However it is assumed that they can affect the post-transcriptional processing of mRNA by interfering with the process of removing introns or affect the process of alternative transcript splicing. What’s more, silent polymorphisms can be important in the process of protein folding, leading to its abnormal form. In addition, as Kimchi-Sarfaty *et al*.^11^ indicate, replacing as a result of silent polymorphisms often used in translation of codons into rare ones can affect the rate of protein folding, and thus change its function or change its substrate specificity. On the other hand, synonymous polymorphisms can change the structure and/or function of a protein by coupling to non-synonymous polymorphisms that directly change the amino acid sequence of a protein^11–15^.

P-gp is expressed in the apical membranes of many tissues and can be implicated in numerous various processes like differentiation, proliferation, apoptosis and immune response regulation^16^. In a normal lung, P-gp is expressed on the top surface of the bronchial epithelium, where it can act to remove external compounds from the lung. In lung cancer, initially low P-gp expression level, can change after exposure to chemotherapy as part of acquired drug resistance. P-gp confers resistance to cytotoxic drugs, including etoposide and cisplatin, and polymorphisms may affect the specificity of the substrate^17^. Research on the influx and efflux mechanisms of drug transporters may be useful to assess the effectiveness of therapy. Various studies have shown that the family of ATP-binding transport proteins (ABC transporters), such as ABCB1 or ABCG2, may be associated with the development of drug resistance^18^. On the other hand, primary decreased P-gp expression may be associated with accumulation of the metabolites or drugs and as a consequence diseases development.

This study aimed to evaluate the association between the SNP 3435 and the expression of the *ABCB1* gene in lung cancer patients in the Polish population in comparison to clinicopathological parameters and treatment.

## Results

### Genotyping of C3435T of the *ABCB1* gene

47 blood samples collected from patients with lung cancer for SNP on position C3435T of the *ABCB1* gene were successfully analyzed, for two samples no PCR product was obtained. The polymorphism in both lung cancer patients and healthy individuals were in Hardy-Weinberg equilibrium (Table 1).

**Table 1:** Frequencies of the C3435T *ABCB1* gene genotypes in lung cancer patients and healthy individuals.

*ABCB1* C3435T	Lung cancer Patients N = 47	Healthy Individuals N = 96	p (Chi^2 Pearson)	Odds ratio	95% Cl
CC	4 (8.5 %)	27 (28.1 %)	***0.0124***	1	—
CT	25 (53.2 %)	48 (50.0 %)		3.52	1.11–11.17
TT	18 (38.3 %)	21 (21.9 %)		5.78	1.70–19.68
C	33 (35.1 %)	102 (53.1 %)	***0.0041***		
T	61 (64.9 %)	90 (46.9 %)			
HWE p (Chi^2 Pearson)	0.6527	0.9991			

Firstly, genotype and allele frequencies for the studied polymorphism between the group of patients with lung cancer and the group of healthy individuals were compared. For SNP at position C3435T, the genotype TT (mutant homozygous) and allele T were statistically significantly more frequent in the group of patients with lung cancer than in the control (*p* = *0.0124; p* = *0.0041*, respectively). All data is summarized in Table 1.

Secondly, the lung cancer patients were divided into patients under and equal 67 years old and over 67 years old (average age of the group), and frequencies of SNP C3435T genotypes were compared. No statistical significance was found (*p* = *0.6578*).

Thirdly, the group of patients with cancer was divided according to gender into subgroups of women and men. In this case, the TT genotype tended to be more frequent in the subgroup of men with lung cancer (the subgroup of females TT 10%; the subgroup of men 46%; *p* = *0.0706*).

Next, the investigated group was divided according to their histological type into subgroups of patients with squamous cell carcinoma and adenocarcinoma. The TT genotype (mutant) occurred more frequently in the group of patients with squamous cell lung carcinoma than in the subgroup with lung adenocarcinoma (TT 46.4%; TT 26.3%, respectively). However, no statistical significance was found (*p* = *0.2252*).

Also in the group of patients with lung cancer, based on their medical history, the subgroup of patients who smoked tobacco and a subset of those who did not were listed. There was a tendency for the TT genotype to be more frequent in the subset of tobacco smoking patients (TT 50%) than in the subgroup of non-smokers (TT 17.6%) (*p* = *0.0902*).

After that, dependencies between clinicopathological parameters (TNM stage and grade of histological malignancy) and the C3435T polymorphism in the group of lung cancer patients were verified. Investigated group was divided according to the TNM classification into two subgroups of patients: with less (IA1 or IA2 or IB) and with more advanced clinical stage (IIA or IIB or IIIA). The genotype frequency was then compared between the two subgroups. TT genotype occurred more frequently in patients with less advanced clinical stage (47.8%) than in the group of patients with more advanced clinical stage of cancer (29.2%). No statistically significant difference was observed (*p* = *0.3929*), though. Then, according to the histological malignancy grade, the investigated cohort was divided into a subgroup with G1 and G2 grades combined together and a subgroup of patients with a G3 grade. There was a tendency for the TT genotype to occur more frequently in the subgroup of patients with a G1 or G2 grade than in the subgroup of patients with a G3 grade (TT 42.9%; TT 25%; *p* = *0.0517*). All this data is summarized in Table 2.

**Table 2:** Frequency of genotypes and alleles of the SNP C3435T *ABCB1* gene according to clinicopathological parameters.

		*ABCB1* C3435			p
		CC	CT	TT	
		N (%)			
Gender	Women	2 (20)	7 (70)	1 (10)	*0.0706*
	Men	2 (5.4)	18 (48.6)	17 (46)	
Tobacco smoking	Non-smokers	2 (11.8)	12 (70.6)	3 (17.6)	*0.0902*
	Smokers	2 (6.7)	13 (43.3)	15 (50)	
Histological type of cancer	squamous cell carcinoma	3 (10.7)	12 (42.9)	13 (46.4)	0.2252
	adenocarcionma	1 (5.3)	13 (68.4)	5 (26.3)	
TNM stage	IA1 or IA2 or IB	2 (8.7)	10 (43.5)	11 (47.8)	0.3929
	IIA or IIB or IIIA	2 (8.3)	15 (62.5)	7 (29.2)	
Grade of histological malignancy [G]	G1 or G2	1 (2.8)	19 (54.3)	15 (42.9)	*0.0517*
	G3	3 (25)	6 (50)	3 (25)	

The dependence of the genotype on polymorphism at position C3435T of the *ABCB1* gene on probability of overall survival time

**Figure 1:**
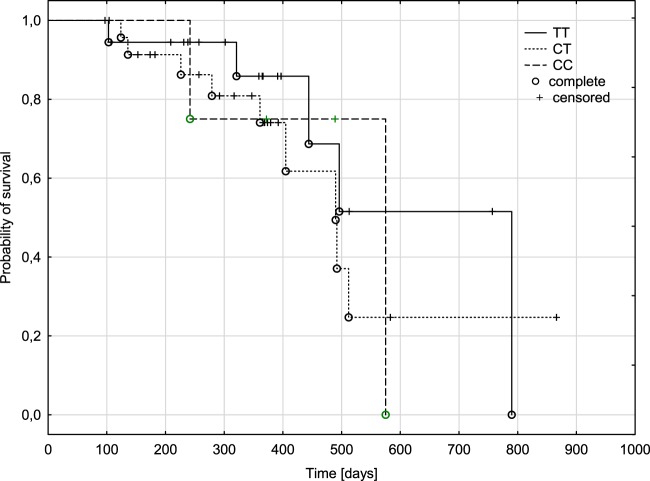
Overall survival plots for lung cancer patients with different genotypes for C3435T polymorphism of the *ABCB1* gene.

The Kaplan-Meier plot shows the probability of survival in the group of patients with lung cancer from the time of cancer diagnosis up to over 2 years of observation of the patients (Fig. 1). Median survival time was shorter in patients with CT (347 days) or TT (362 days) genotype compared to CC genotype (430 days). However, there was no statistical significance difference in overall survival time according to C3435T genotypes (p = 0.5999).

### Expression of the *ABCB1* gene in blood patients with lung cancer

Relative expression level of the *ABCB1* gene was successfully analysed in the same group of patients, blood samples were collected at three points of time.

The mRNA was isolated and its level was analysed in 40 blood samples collected at the time of lung cancer diagnosis, 39 samples 100 days after the surgery and 24 from patients one year after resection (the group is smaller because patients were lost from observation or died). The obtained results were compared with the control group of 56 blood samples from people without cancer. The first comparison involves changing of the relative expression of the *ABCB1* gene between all investigated groups: control, patients at the time of diagnosis, 100 days after the surgery and one year after the surgery.

**Figure 2:**
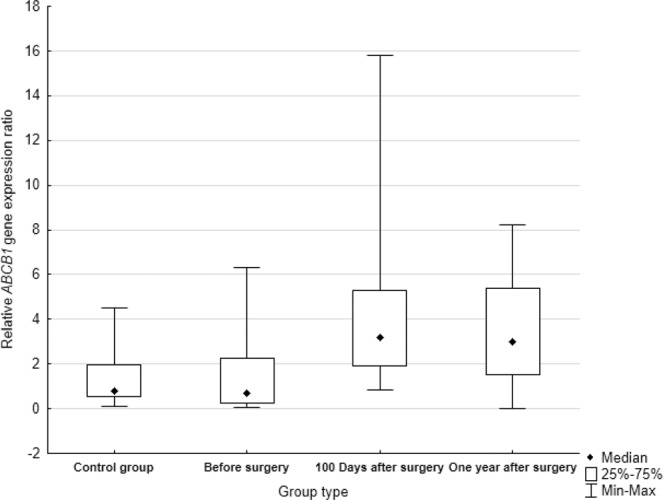
Relative expression levels of *ABCB1* in control group and blood taken from patients at three points during the diagnostic-therapeutic procedure.

The obtained results showed statistically significant differences in the expression of the *ABCB1* gene in the group of patients 100 days after the surgery (*p* = *0.0000*) and one year after the surgery (*p* = *0.0000*) compared to the control group. No statistically significant differences were found for the group of patients at the time of diagnosis compared to control (*p* = *0.3049*). In addition, a statistically significant increased *ABCB1* mRNA level was found in the group of patients 100 days after the surgery and one year after the surgery in comparison to samples at the time of the diagnosis (*p* = *0.0000* and *p* = *0.0081*, respectively). However, there were no differences in the *ABCB1* gene expression between the groups of patients 100 days after the surgery and one year after the surgery (*p* = *0.6622*). Data is summarized in Fig. 2.

In the group of patients, correlation between relative mRNA of *ABCB1* expression and age of patients was assessed. No statistically significant correlation was found (*p* = *0.315*). After that, patients were divided according to gender. In this case, also no statistical significance between the subgroup of women and men was found (*p* = *0.7738*).

Next, the lung cancer cohort was divided according to histological type of cancer into the patients with squamous cell lung carcinoma (N = 21) and with adenocarcinoma (N = 16). There was no statistically significant difference in relative *ABCB1* gene expression between these two subgroups (*p* = *0.1453*).

The group of patients with lung cancer was also divided into the subgroup of tobacco smokers (N = 25) and non-smokers (N = 14). Also in this analysis, no statistically significant differences between *ABCB1* mRNA expression level and tested subgroups were found (*p* = *0.4553*).

**Figure 3:**
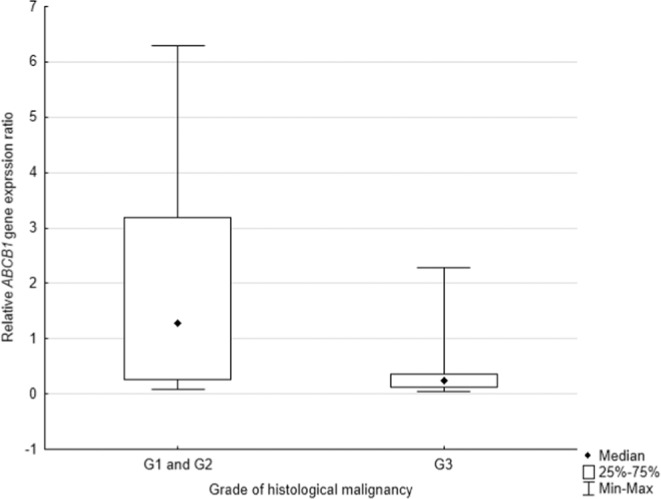
The level of *ABCB1* expression in comparison to histological grade of malignancy.

Then, the group of patients with cancer was divided according to grade of histological malignancy into subgroups of: highly differentiated cancer - G1 or moderate grade - G2 patients and poorly differentiated - G3 patients. Due to a small number of patients who were classified into G1 group (N = 3) the combined G1 and G2 (N = 27) groups with G3 (N = 9) were compared. The analysis showed a lower *ABCB1* gene expression in patients with poorly differentiated cancer cells than in patients with G1 and G2 stage (*p* = *0.0352*) Fig. 3.

**Figure 4:**
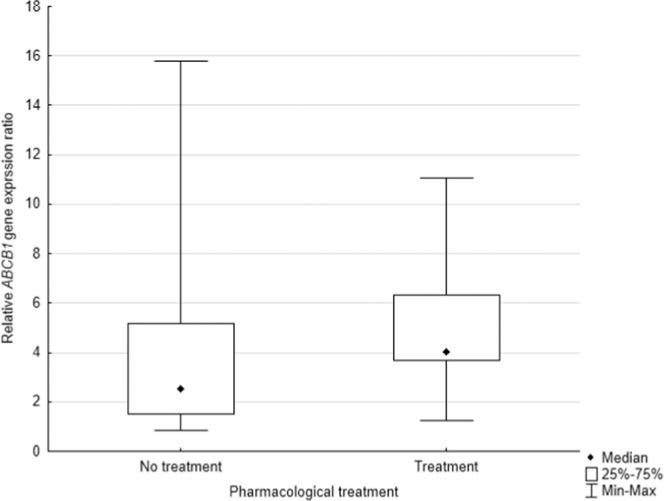
The level of *ABCB1* expression in comparison to the scheme of treatment.

**Figure 5:**
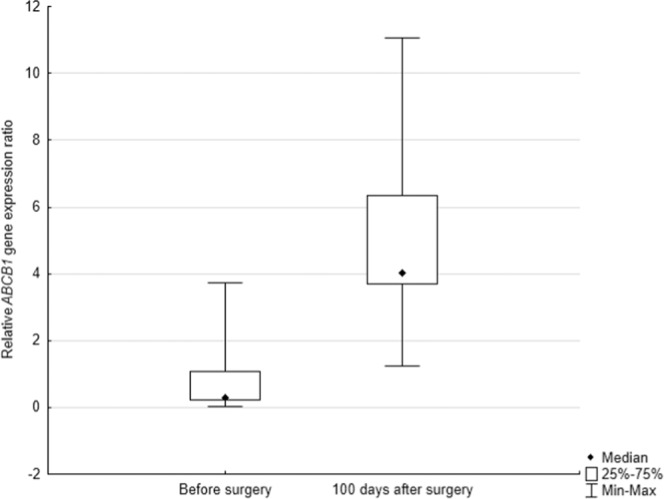
The level of *ABCB1* expression in the subgroup of patients who received adjuvant chemotherapy treatment (*p* = *0.0094*).

In the next step, the expression of the *ABCB1* gene was evaluated according to the use of additional pharmacological treatment after the surgery. Two groups were distinguished, the first one received various types of complementary pharmacological treatment and revealed expression of *ABCB1* (N = 12; Table 3), the second with only surgical intervention (N = 27). The results of the conducted analysis showed a statistically significant difference in expression of *ABCB1* in people who received pharmacological treatment during 100 days observations between this two groups. (*p* = *0.0428*), Fig. 4. Additionally, the *ABCB1* gene expression level was evaluated in the subgroup of patients who received chemotherapy treatment, the analysis also revealed the statistical significant difference in *ABCB1* gene expression level. Patients before surgery have lower *ABCB1* gene expression than 100 days after surgery and pharmacological treatment (p = 0.0094; Fig. 5). The third time point (one year after surgery) was not included since the data was limited to only five patients.

**Table 3:** Scheme of applied chemotherapy in the group of patients with *ABCB1* expression.

Scheme of chemotherapy	Number of patients
carboplatin + gemcitabine	2
cisplatine + etoposide	2
etoposide	2
cisplatine + vinorelbine	6

In further analysis, no significant differences were found among relative *ABCB1* gene expression and clinicopathological features like distant metastases (*p* = *0.2725*), the size of primary tumor (*p* = *0.9841*), the involvement of local lymph nodes (*p* = *0.7493*), leukocyte count (*p* = *0.7753*). However, a statistically significant decreased expression of the *ABCB1* gene was observed in patients with fibrinogen concentration above the range of reference values (*p* = *0.04943*).

### Expression of the *ABCB1* gene in lung cancer tissue

In the same group of patients the *ABCB1* gene expression was also examined in 47 tumor tissues taken during the surgical procedure and compared to the expression level in the blood before the surgery and then to clinical-pathological features. There were no statistically significant differences between the level of *ABCB1* expression in tissue and blood before the surgery (*R* = *−0.0138*). Also there were no dependences between the expression of *ABCB1* gene cancer altered tissue and clinical-pathological features: tobacco smoking (*p* = *0.0627*), histological type (*p* = *0.6137*), grade of histological malignancy (*p* = *0.1223*), the involvement of local lymph nodes (*p* = *0.1446*), gender (*p* = *0.7797*) and age (*R* = *−0.014*).

On the basis of our study we can conclude that mutant homozygous TT and allele T are present significantly more frequent in the group of patients with lung cancer nevertheless are connected with more differentiated tumors (G1/G2). There is no difference with the expression level among lung cancer tissue and blood sample taken from the same patients before surgical treatment. On the basis of blood samples analysis it was observed that the expression level of *ABCB1* mRNA was increasing in time, higher levels were determined after 100 days and one year from the surgical intervention. The complementary pharmacological treatment induced higher expression levels which could preliminary suggest the development of multidrug resistance mechanism.

## Discussion

In the present study, the incidence of the genotype at position C3435T of the *ABCB1* gene in both the test and control groups were consistent with the Hardy-Weinberg equilibrium. The distribution of genotypes prevalence was similar to that in the Caucasian, German-Caucasian or French populations, and different than in the African population, where the CC genotype predominates^19–22^.

P-glycoprotein, encoded by *ABCB1* gene, is present in many normal tissues, including bronchi and lungs. It plays a protective role where, by moving xenobiotics to the extracellular environment, it protects cells from its toxic/carcinogenic activity cell-toxins^23,24^.

The *ABCB1* gene is polymorphic and it has been shown that several of these polymorphisms may be linked with the functioning of P-glycoprotein. Similarly, the occurrence of polymorphisms may be linked with increasing predisposition to various diseases development, including cancer. One of these is polymorphism at position C3435T of the *ABCB1* gene, where Hoffmayer *et al*. have shown that it may change the expression of *ABCB1* mRNA and P-glycoprotein function^25^.

The aim of this study was to assess the potential impact of the *ABCB1* gene on the risk of non-small cell lung cancer development. To the best of our knowledge, this is the first such study in the Polish population. To date, most of the worldwide studies on the C3435T polymorphism and lung cancer have focused on the effectiveness of the therapy and not on the risk of developing this cancer.

In this study, by comparing the frequency of genotypes and alleles occurrence of a given polymorphism between the group of patients with non-small cell lung cancer and the control group, it was shown that the TT genotype and allele T of the *ABCB1* gene C3435T were significantly more frequent in the group of cancer patients (*p* = *0.0124; p* = *0.0041*, respectively). This indicates that the presence of at least one T allele of polymorphism at the C3435T position of the *ABCB1* gene is associated with an increased risk of developing non-small cell lung cancer. Additionally, the risk of developing lung cancer was 5.78 times higher in the presence of the TT genotype of the studied polymorphism. These results are similar to those obtained by Subhani *et al*. where TT genotype of SNP 3435 was associated with 5.23-fold higher risk of lung cancer development^26^. The obtained results are in contrast to those obtained by Sinues *et al*. and Gervasini *et al*., where the relationship between polymorphism at position C3435T and the risk of developing lung cancer has not been demonstrated^27,28^. The increased efflux of rhodamine 123 from CD56 cells^29^ may be related to the loss of the protective function of the P-glycoprotein as indicated by Hiltz *et al*. in the genotype CC of SNP 3435.

Thus, the presence of the TT genotype for this SNP will be associated with the intracellular accumulation of xenobiotics with a potential carcinogenic activity. It is worth noting that the presented study showed a tendency to more frequent occurrence of the TT genotype in the subgroup of people with lung cancer who smoked tobacco compared to the subgroup of non-smokers (*p* = *0.0902*). However, other studies show no association between tobacco smoking and SNP 3435 of *ABCB1* gene^27,28^.

The assessment of genotypes’ frequencies in the group of patients with lung cancer of different clinical stage of cancer (TNM stage) and the grade of histological malignancy of cancer (Grading) allowed to determine the importance of C3435T polymorphism in progression of gastric cancer. Due to the fact that there were no statistically significant correlations between less advanced clinical stage of cancer and patients with more advanced clinical stage of cancer (*p* = *0.3929)* no association between SNP 3435 and the clinical stage of the cancer was demonstrated, which was in contrast to Subhani *et al*. who showed that TT genotype of C3435T was associated with the advanced stage of lung cancer^26^. On the other hand, in this study, the TT genotype in a subset of patients with G1 or G2 histologic malignancy of lung cancer (*p* = *0.0517*) tends to occur more frequently, which is additionally confirmed by the result of the study for the mRNA expression of the *ABCB1* gene, where it is demonstrated that the expression of mRNA is higher in the subgroup of patients with G1 or G2 (*p* = *0.0278*). This dependence is confirmed by the results of the Subhani *et al*. who also showed the correlation between the expression of *ABCB1* and the intermediate degree of histological malignancy of the cancer^26^.

However, we did not confirm an observation noted by other authors that during cancer development the expression level is decreased which can be connected with the loss of physiological, protecting function of encoded by the *ABCB1* gene protein. Delou *et al*. reported the loss of constitutive *ABCB1* expression in breast cancer, especially in triple-negative tumors that seems to indicate a subgroup of a worse prognosis^30^. Our data did not show the differences between the expression level in blood and tissue samples in comparison to control group, also with clinicopathological parameters known as a worse prognostic and predictor factors.

In our study, the expression of the *ABCB1* gene was checked in cancer tissue and in blood samples taken from patients at three points (at the time of diagnosis, 100 days and one year from the surgical intervention). Data was compared to each other and to the control group. A tendency for the expression level to increase in time was observed after 100 days and one year after the operation, the levels of mRNA were higher in a group to whom additional chemotherapy was administered, which can be related to developing a multidrug resistance process, similar observation was noted by Weissfeld^31^. This is in agreement with a higher frequency of TT genotype in our investigated group.

The impact of *ABCB1* C3435T polymorphism on the function of P-gp can be explained by many hypotheses concerning the influence of a silent polymorphism on features and predispositions revealed phenotypically. One of them assumes the influence of a SNPs on the translation effectiveness. It is also possible that differences in allele specific present in RNA secondary structure could change the splicing process or the translation control. Another one assumes that some of polymorphisms increase the mRNA stability, which in consequence leads to the increased protein level and/or a change of the substrates’ affinity to the P-gp transporter. The modified function of P-gp could be a risk and progression factor of lung cancer^32^.

We are aware of the limitations of our study, particularly in the restriction of investigated cases number, especially after their classification according to clinical-pathological parameters the resulting groups were small, nonetheless, it still allowed us to perform a statistical analysis. The convergence of the results obtained in post-operative tissues and blood trials should be emphasized.

In our ongoing project, we would like to investigate two other polymorphisms, one leading to amino acid exchange (*ABCB1* G2677T/A) and the other one, which has no influence on the amino acid sequence of P-gp but surprisingly, may influence the P-gp function (*ABCB1* C1236T). With data on several polymorphisms, it is possible to conduct the haplotype analysis which may provide more useful information than the genotype in case of only one polymorphism^16^. In our previous study it was proven that the three investigated SNPs of the *ABCB1* gene (*ABCB1* C1236T, *ABCB1* G2677T/A and *ABCB1* C3435T) are located in one haploblock^32^.

The presented data suggests an important role of *ABCB1* in lung cancer which we would like to prove in our future research.

## Materials and Methods

### Investigated group

The investigated group comprised of 49 patients (10 female and 39 male) who were diagnosed with non-small cell lung cancer (squamous cell carcinoma and adenocarcinoma) at the N. Copernicus Regional Specialist Hospital in Lodz, Poland. The mean age at the time of diagnosis was 67.1 years (64.4 for females and 67.8 for males). Peripheral blood of selected patients collected between 2016–2018 was used for research. Samples were collected at three time points: at the time of cancer diagnosis, 100 days after the surgery and one year after the surgery. 47 patients from the entire group underwent a surgical resection, frozen tissue sections were additionally obtained from these patients. In 15 cases adjuvant chemotherapy was included after the surgery (carboplatin + gemcitabine 2, cisplatine + etoposide 2, etoposide 2, cisplatine +vinorelbine 9).

For genotyping at position C3435T of the *ABCB1* gene the DNA was successfully isolated from 49 blood samples collected at the time of diagnosis of cancer.

For the expression level analysis of the *ABCB1* gene the RNA was successfully isolated from:

- 40 blood samples collected at time of diagnosis of cancer
- 39 blood samples collected 100 days after the surgery
- 24 blood samples collected one year after the surgery
- 47 frozen tissue sections collected intraoperatively

### Control group

The group of healthy individuals (control group) consisted of 96 blood donors from the local blood bank, geographically and ethnically matching the group of patients with non-small cell lung cancer. 96 DNA and 56 RNA samples were successfully isolated from peripheral blood.

The investigation was in accordance with the principles of the Declaration of Helsinki and was approved by the Ethical Committee of the Medical University of Lodz (No RNN/87/16/KE). All individuals included in the study gave their informed consent.

### DNA and RNA isolation

DNA and RNA from peripheral blood and frozen tissue sections collected intraoperatively were isolated according to “Blood Mini” and “Total RNA Mini” protocol, respectively (*A&A Biotechnology*, Poland)^33,34^. The purity and concentration of DNA and RNA samples were assessed nanospectrophotometrically. Concentration of extracted DNA samples range from 25 to 50 ng/ul, for RNA range from 5,2–80 ng/ul to obtain concentration for reverse transcriptase reaction described in the next subsection. Until the analysis, the DNA and RNA samples were stored at −20 °C and at −76 °C, respectively.

### Genotyping C3435T of *ABCB1* gene

#### Polymerase Chain Reaction (PCR)

For studied polymorphism a polymerase chain reaction (PCR) was performed in accordance with 2xPCR Super Master Mix (*Biotool.com, USA*) protocol. The mixture for PCR reaction consisted of 5 μl of 2xPCR Super Master Mix; 0.5 mM of each primer (Forward 5′TTGATGGCAAAGAAATAAAGC3‘ and Reverse 5′CTTACATTAGGCAGTGACTCG3‘) specific to particular SNP; 50 ng of DNA template and distilled water up to 20 μl. Negative control was included in every experiment. Products of the PCR reactions were assessed using electrophoresis in 2% agarose gel. Reaction products for SNP at position 3435 was the size of 208 bp^35^.

#### Restriction Fragment Length Polymorphism (RFLP)

Amplified DNA fragments for SNP on position 3435 were digested by *MboI* (EURx, Poland) for 16 h at 37 °C. Genotypes were identified using electrophoresis of amplified DNA fragments after digestion by restriction enzyme (one band of 208 bp for genotype TT; two bands of 145 and 63 bp for genotype CC; three bands of 208, 145 and 63 bp for genotype CT).

The results of *ABCB1* genotyping of the healthy individuals were described previously^36^.

### Expression of *ABCB1 mRNA*

#### Reverse transcription

A total cellular RNA was transcribed into complementary DNA (cDNA) in accordance with High-Capacity cDNA Reverse Transcription Kit protocol (*Applied Biosystems; USA*)^37^. The final concentration of RNA in reaction mixture was 0.005 μg/μl. Synthesized cDNA were stored at −20°C until analysis. As reference the *GAPDH* gene, encoding glyceraldehyde-3-phosphate dehydrogenase, was used^38^. Only the samples which showed the presence of PCR product for the *GAPDH* gene (188 bp) were included in further analysis.

### Real – time polymerase chain reaction (real-time PCR)

Quantification assessment of *ABCB1* (the investigated gene) and *GAPDH* (the reference gene) mRNA was performed in real-time PCR using the Rotor-Gene 6000 (*Corbet Research, Germany*) according to “SYBRGreen JumpStart *Taq* ReadyMix” protocol^39^. The reaction mixture for both genes consisted of 7.5 μl SYBR-Green ReadyMix, 0.7 μl of each primer (*ABCB1* gene: F 5′-GGCCTAATGCCGAACACATT-3′, R 5′-CAGCGTCTGGCCCTTCTTC-3′; *GAPDH* F 5′-TGGTATCGTGGAAGGACTCAT-3′, R 5′-ATGCCAGTGAGCTTCCCGTTCAGC-3′), 1 μl of cDNA and distilled water up to 16 μl final volume. The reactions for *ABCB1* and *GAPDH* were carried out in separate tubes. Samples were tested in triplicates and mean of obtained Ct values for both *ABCB1* and *GAPDH* was calculated. In each experiment, negative control, also tested in triplicates, was included. To calculate relative changes in the gene expression, the ΔΔ C_t_ method was used^40^.

### Statistical analysis

All statistical analyses were performed using STATISTICA 13 (*StatSoft Inc.2018*). The Chi^2 Pearson test was applied to evaluate conformity between the observed and expected genotype frequencies according to Hardy-Weinberg rule. To determine the significance of differences in allele and genotype frequencies between the group of lung cancer patients and the group of healthy individuals, as well as analysis of patients data (age, gender, tobacco smoking and clinical and pathological factors) in the lung cancer individuals Chi^2 Pearson was used. To determine the validity between the R-value and genotypes, age, gender, tobacco smoking or clinical and pathological factors the U Mann – Whitney test was used. The Kaplan-Meier analysis was done to estimate overall survival time^41,42^. In all conducted tests a *p* value of < 0.05 was assumed as significant.

### Ethics approval and consent to participate

The present study was approved by The Ethics Committee of the Medical University of Lodz (No RNN/87/16/KE) and was in accordance with the principles of the Declaration of Helsinki. Written informed consent was obtained from the patients prior to their participation in the research.

## Data Availability

Correspondence and requests for materials should be addressed to E.B.
